# Association between Lipoprotein(a) and diabetic nephropathy in patients with type 2 diabetes

**DOI:** 10.3389/fendo.2023.1337469

**Published:** 2024-01-15

**Authors:** Meng Li, Yanjun Wang, Qianqian Yao, Qian Liang, Yuanyuan Zhang, Xin Wang, Qian Li, Wei Qiang, Jing Yang, Bingyin Shi, Mingqian He

**Affiliations:** ^1^ Department of Endocrinology, The First Affiliated Hospital of Xi’an JiaoTong University, Xi’an, Shaanxi, China; ^2^ Med-X Institute, Center for Immunological and Metabolic Diseases, The First Affiliated Hospital of Xi’an JiaoTong University, Xi’an, Shaanxi, China

**Keywords:** type 2 diabetes mellitus, diabetic nephropathy, dyslipidemia, Lipoprotein(a), renal impairment

## Abstract

**Background:**

Diabetic nephropathy (DN) is one of the most prevalent and severe microvascular complications of type 2 diabetes (T2DM). However, little is currently known about the pathogenesis and its associated risk factors in DN. The present study aims to investigate the potential risk factors of DN in patients with T2DM.

**Methods:**

A total of 6,993 T2DM patients, including 5,089 participants with DN and 1,904 without DN, were included in this cross-sectional study. Comparisons between the two groups (DN vs. non-DN) were carried out using Student’s t-test, Mann-Whitney U-test, or Pearson’s Chi-squared test. Spearman’s correlation analyses were performed to assess the correlations of serum lipids and indicators of renal impairment. Logistic regression models were applied to assess the relationship between blood lipid indices and the presence of DN.

**Results:**

T2DM patients with DN were older, and had a longer duration of diagnosed diabetes compared to those without DN. Of note, the DN patients also more likely develop metabolic disorders. Among all serum lipids, Lipoprotein(a) [Lp(a)] was the most significantly correlated indicators of renal impairment. Moreover, univariate logistic regression showed that elevated Lp(a) level was associated with an increased risk of DN. After adjusted for confounding factors, including age, gender, duration of T2DM, BMI, SBP, DBP and lipid-lowering drugs usage, Lp(a) level was independently positively associated with the risk of DN [odds ratio (OR):1.115, 95% confidence interval (CI): 1.079-1.151, *P*=6.06×10^-11^].

**Conclusions:**

Overall, we demonstrated that serum Lp(a) level was significantly positively associated with an increased risk of DN, indicating that Lp(a) may have the potential as a promising target for the diagnosis and treatment of diabetic nephropathy.

## 1 Introduction

The prevalence of diabetes mellitus (DM) has increased rapidly worldwide, mostly driven by an increase in the prevalence of type 2 diabetes mellitus (T2DM). Consequently, the global prevalence of microvascular and macrovascular complications associated with DM is increasing dramatically (1). Among them, diabetic nephropathy (DN) is one of the most prevalent and severe microvascular complications of DM, with an incidence of approximately 20% in patients with T2DM (1, 2), and DN has become the leading cause of chronic kidney disease or even end-stage renal disease worldwide (3, 4). In addition, DN can significantly increase cardiovascular morbidity and mortality and decrease the health-related quality of life for patients (5). However, the pathogenesis of DN remains poorly understood, and once in the clinical phase it is difficult to reverse, placing a substantial burden on the public health and the social economy (6). Therefore, there is an urgent need to improve the understanding of DN and its associated risk factors, and then contribute to the prevention and management of DN, thereby improving the prognosis and quality of life of diabetics.

Diabetes complications, including DN, are associated with lipid metabolism disruption and lipid accumulation. Several previous evidence support that hyperlipidemia may related to the occurrence of kidney disease, also in DM. Lipoprotein(a) [Lp(a)], a low-density lipoprotein-like particle consisting of apolipoprotein A (ApoA) bounding covalently to apolipoprotein B (ApoB)-100, has been well considered as a critical risk factor of cardiovascular disease due to its atherogenic effects (7). Atherosclerosis is a major complication associated with DN, and previous studies have attempted to elucidate the possible role of Lp(a) in cardiovascular disease (8, 9). Studies of the relationship between Lp(a) level and the occurrence of DN in T2DM patients have yielded inconsistent results. Previous studies have revealed that serum Lp(a) level increased gradually with the progression of DN stage among 90 patients with T2DM in Korea (10) and Lp(a) level may be positively associated with DN (11, 12). A meta-analysis included 9,304 T2DM patients from 11 observational studies, and found that higher serum Lp(a) was associated with higher odds of DN (13). In contrast, other studies found that elevated Lp(a) level had no significant effects on other T2DM microvascular complication (14, 15), and also had no effects on the occurrence of DN among 516 women with T2DM in America (16), probably due to the small number of samples investigated. At present study, we comprehensively demonstrated the association between Lp(a) and DN in Chinese T2DM patients through a large sample cross-sectional study.

## 2 Methods

### 2.1 Study design and participants

In this study, inpatients diagnosed with diabetes according to the diagnostic criteria were enrolled in the Department of Endocrinology at the First Affiliated Hospital of Xi’an Jiaotong University. The subjects were excluded if they met any of the following criteria: (1) other types of diabetes other than T2DM or recent acute complications of diabetes; (2) the existence of major diseases or related diseases, such as inflammatory disease, rheumatologic disease, adrenal disease, malignancy, cirrhosis, chronic kidney disease, acquired immunodeficiency syndrome. All patients were assessed by a professional clinician to determine if he or she had a diagnosis of DN based on international diagnostic criteria. Finally, a total of 6,993 patients were invited to participate in our study. All participants signed informed consent with full knowledge of the study protocol. The study was approved by the Medical Ethics Committee of the First Affiliated Hospital of Xi’an Jiaotong University.

### 2.2 Data collection

The study collected basic information, anthropometric measurements, medication history, and laboratory tests from these hospitalized patients through the electronic medical record system. Basic information included age, gender, type and duration of diabetes, and presence of DN. Anthropometric measurements included body mass index (BMI), systolic blood pressure (SBP), diastolic blood pressure (DBP). Medication history included whether the patient was taking insulin, oral hypoglycemic drugs [including biguanides, sulfonylureas, gluconides, alpha-glucosidase inhibitors, thiazolidinediones, dipeptidyl peptidase 4 (DPP4) inhibitors, and sodium-glucose cotransporter 2 (SGLT-2) inhibitors], glucagon-like peptide 1 (GLP-1) analogues and lipid-lowering drugs, etc. Laboratory tests included glycosylated hemoglobin A1c (HbA1c), kidney function, serum lipids, urinary microalbumin creatinine ratio, etc. All laboratory tests were measured following an 8-h overnight fast for each participant and were routinely carried out in the hospital clinical laboratory using standard assays to ensure that measurement errors rarely occurred.

### 2.3 Statistical analysis

Statistical analyses were performed using R version 4.1.3. A two-tailed p value of less than 0.05 was considered statistically significant. Tests for normality were conducted. All data are presented as the mean ± standard deviation (SD) for normally distributed variables or median (interquartile range, IQR) for abnormally distributed variables. Comparisons between the two groups (DN vs non-DN) were carried out using Student’s t-test, Mann-Whitney U-test, or Pearson’s Chi-squared test, and the P values for trend were corrected by false discovery rate (FDR) to reduce the risk of type I error in the statistical tests. Spearman’s correlation analyses were performed to assess the correlations of serum lipids and indicators of renal impairment. Logistic regression models were applied to assess the relationship between blood lipid indices and the presence of DN. The area under the curve (AUC) of receiver operating characteristic curve (ROC) was used to calculate the discriminatory performance for DN presence in ROC analysis.

## 3 Results

### 3.1 Clinical characteristics of the study participants

A total of 6,993 patients with T2DM were recruited in the study, including 5,089 patients with DN and 1,904 patients without DN. The median age of all participants was 57 years (IQR: 48-65 years), and 64.2% (4,490) were males.

As shown in **Table 1**, as compared to T2DM patients without DN, T2DM patients with DN were older, had a longer duration of diagnosed diabetes, and the higher proportion of insulin and lipid-lowering drugs usage. In addition, the DN patients had higher SBP, DBP, BMI, levels of HbA1c and blood glucose, with all *P* value < 0.05, indicating a greater potential burden of metabolic disorders. Meanwhile, the renal function was deteriorated in patients with DN, as shown by higher glycated albumin, uric acid, blood glucose, cystatin C, blood creatinine, blood urea nitrogen, urinary albumin creatinine ratio (UACR), 24-hour urine protein (24hU-TP), microalbumin, 24-hour microalbumin and lower estimated glomerular filtration rate (eGFR), urine creatinine (all *P* value < 0.05). Of note, compared with non-DN patients, DN patients had higher levels of serum apolipoprotein E (ApoE), ApoB/ApoA, Lp(a), triglyceride (TG), and higher ratio of TG/high-density lipoprotein cholesterol (HDL-C) and low-density lipoprotein cholesterol (LDL-C)/ApoB, with all P value < 0.05, indicating the disturbance of lipid metabolism in DN.

**Table 1:** Clinical characteristics of the study participants.

	None-DN	DN	*P* value*
	(N=5089)	(N=1904)	
Basic information			
Male (%)	3232 (63.5%)	1258 (66.1%)	0.050
Age (years)	54.50 ± 14.32	59.53 ± 12.34	**<0.001**
Duration (years)	7.31 ± 6.72	12.04 ± 7.43	**<0.001**
Medication history			
Insulin	3095 (60.8%)	1551 (81.5%)	**<0.001**
Oral hypoglycemic drugs	3510 (69.0%)	971 (51.0%)	**<0.001**
GLP-1 analogues	182 (3.6%)	59 (3.1%)	0.368
Lipid-lowering drugs	3934 (77.3%)	1611 (84.6%)	**<0.001**
Anthropometric measurements			
SBP (mmHg)	130.30 ± 17.47	141.12 ± 21.68	**<0.001**
DBP (mmHg)	79.24 ± 10.82	81.94 ± 12.43	**<0.001**
BMI (kg/m^2^)	24.52 ± 3.71	24.65 ± 3.56	**0.040**
HbA1c (%)	8.85 ± 2.40	8.98 ± 2.21	**0.002**
Kidney function			
Glycated albumin (%)	23.88 ± 8.86	24.37 ± 9.90	**0.049**
Uric acid (umol/L)	316.4 ± 126.9	341.7 ± 160.1	**<0.001**
eGFR (mL/min/1.73m^2^)	107.15 ± 19.53	90.16 ± 29.49	**<0.001**
glucose (mmol/L)	8.28 [6.02-12.5]	9.12 [6.39-13.6]	**<0.001**
Cystain C (mg/L)	0.80 [0.67-0.95]	0.98 [0.79-1.33]	**<0.001**
Blood creatinine (umol/L)	56.0 [46.0-66.0]	65.2 [53.0-89.0]	**<0.001**
Blood urea nitrogen (mmol/L)	5.65 ± 2.33	7.38 ± 3.99	**<0.001**
Urinary microalbumin creatinine ratio			
Urine creatinine (umol/L)	7670 [4640-11900]	5750 [3830-8920]	**<0.001**
UACR (mg/g)	11.9 [7.26-20.9]	151 [50.9-674]	**<0.001**
24-hour microalbumin (mg/24h)	15.1 [9.03-25.8]	157 [60.0-810]	**<0.001**
24hU-TP (g/24h)	0.05 [0.03-0.08]	0.26 [0.10-1.23]	**<0.001**
24-hour urine output (ml)	2000 [1400-2600]	2000 [1400-2600]	0.372
Microalbumin (mg/L)	1.80 [0-10.8]	25.1 [0.150-177]	**<0.001**
Serum lipids			
ApoE (mg/L)	33.5 [26.5-43.9]	34.6 [26.8-46.9]	**0.015**
ApoB (g/L)	0.82 ± 0.23	0.85 ± 0.27	0.139
ApoA (g/L)	1.12 ± 0.22	1.11 ± 0.25	0.658
ApoB/ApoA	0.732 [0.584-0.904]	0.748 [0.596-0.935]	**0.024**
Lp(a) (mg/dL)	1.09 [0.56-2.19]	1.41 [0.64-2.96]	**<0.001**
TC (mmol/L)	4.16 ± 1.12	4.25 ± 1.39	0.563
LDL-C (mmol/L)	2.46 ± 0.85	2.50 ± 1.06	0.658
TG (mmol/L)	1.42 [0.98-2.14]	1.45 [1.02-2.30]	**0.024**
HDL-C(mmol/L)	0.98 ± 0.27	0.98 ± 0.31	0.495
TG/HDL-C	1.50 [0.955-2.42]	1.58 [1.02-2.61]	**0.015**
LDL-C/ApoB	3.00 [2.72-3.24]	2.95 [2.63-3.25]	**0.012**
HDL-C/ApoA	0.871 [0.793-0.953]	0.867 [0.786-0.954]	0.563

DN, diabetic nephropathy; GLP-1,glucagon-like peptide 1; SBP, systolic blood pressure; DBP, diastolic blood pressure; BMI, body mass index; HbA1c, glycated hemoglobin; eGFR, estimated glomerular filtration rate; UACR, urinary albumin creatinine ratio; 24hU-TP, 24-hour urine protein; ApoE, apolipoprotein E;Lp(a), Lipoprotein(a); TC, total cholesterol; TG, triglyceride; HDL-c, high-density lipoprotein cholesterol; LDL-c, low-density lipoprotein cholesterol. All data are presented as the mean ± standard deviation (SD) or median and inter quartile range (IQR) for the normally and skewed distributed continuous variables, as well as frequencies and percentages for the categorical variables, respectively. Bold values: significant differences (P value of less than 0.05). Comparisons between the two groups (DN vs non-DN) were carried out using Student’s t-test, Mann-Whitney U-test, or Pearson’s Chi-squared test, and the P values* for trend were corrected by false discovery rate (FDR) to reduce the risk of type I error in the statistical tests.

### 3.2 Serum lipid indices on the occurrence of DN

**Figure 1:**
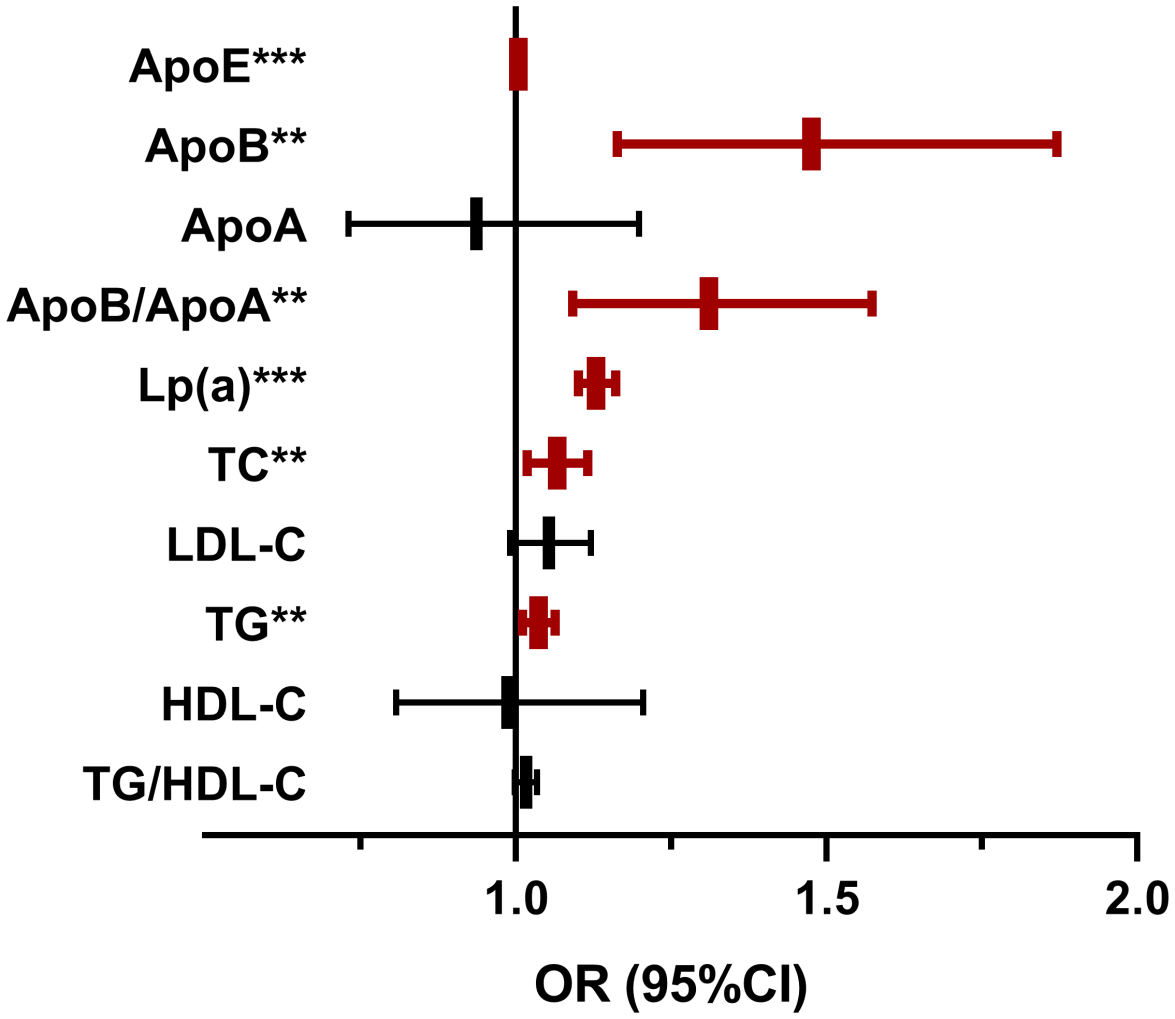
The Lipid Indices on the occurrence of diabetic nephropathy. OR, odds ratio; CI, confidence interval; DN, diabetic nephropathy; ApoE, apolipoprotein E;Lp(a), Lipoprotein(a); TC, total cholesterol; TG, triglyceride; HDL-c, high-density lipoprotein cholesterol; LDL-c, low-density lipoprotein cholesterol. Univariate logistic regression analyses were used to investigate the association of blood lipid indices and DN.

**Figure 2:**
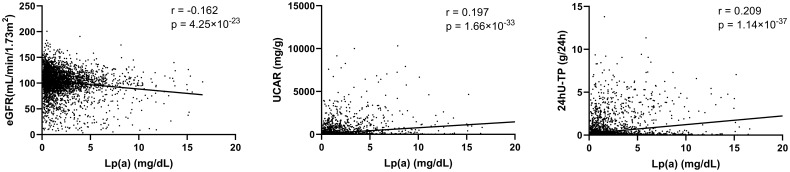
Correlations between Lp(a) and indicators of renal impairment. eGFR, estimated glomerular filtration rate; UACR, urinary albumin creatinine ratio; 24hU-TP, 24-hour urine protein; Lp(a), Lipoprotein(a). Spearman’s correlation analyses were used to analyze the correlations of serum lipids and indicators of renal impairment.

Univariate logistic regression analysis (**Figure 1**) showed that ApoE, ApoB, ApoB/ApoA, Lp(a), TG and TC were positively correlated with the occurrence of DN. We did not find significant association between ApoA, LDL-C, HDL-C, TG/HDL-C, LDL-C/ApoB, HDL-C/ApoA and the risk of DN, with all *P* value >0.05. In order to further investigate the relationships between the above significant blood lipid indices and indicators of renal impairment, we performed the Spearman’s correlation analyses. **Table 2** shows that the correlation coefficient and its significance between blood lipid indices and indicators of renal impairment. It is worth noting that among all serum lipids, the correlations between Lp(a) and indicators of renal impairment were the most significant (**Figure 2**). As Lp(a) was the risk factor for DPN, ROC analysis was further performed, and the result indicated a cut-point value of 1.538 mg/dL (Youden index 0.010; sensitivity 46.98%; specificity 63.00%), with an area under the curve (AUC) of 0.559 (*P*=1.81×10^-12^), 95%CI: 0.542-0.576.

**Table 2:** Correlations between lipid indices and indicators of renal impairment.

	eGFR		UCAR		24hU-TP	
	r	*P*	r	*P*	r	*P*
ApoE	0.053	0.001	0.050	0.002	0.084	3.47×10^-07^
ApoB	0.061	2.21×10^-04^	0.111	1.35×10^-11^	0.148	1.45×10^-19^
ApoA	-0.065	7.75×10^-05^	0.053	0.001	0.051	0.002
ApoB/ApoA	0.069	2.59×10^-05^	0.045	6.03×10^-03^	0.080	1.31×10^-06^
**Lp(a)**	**-0.162**	**4.25×10^-23^**	**0.197**	**1.66×10^-33^**	**0.209**	**1.14×10^-37^**
TC	0.039	0.017	0.107	7.11×10^-11^	0.144	1.49×10^-18^
TG	0.088	9.88×10^-08^	0.020	0.229	0.039	0.018

UACR, urinary albumin creatinine ratio; 24hU-TP, 24-hour urine protein; ApoE, apolipoprotein E; Lp(a), Lipoprotein(a); TC, total cholesterol; TG, triglyceride. Spearman’s correlation analyses were used to analyze the correlations of serum lipids and indicators of renal impairment. Bold values: among all serum lipids, the correlations between Lp(a) and indicators of renal impairment were the most significant.

### 3.3 Multivariate analysis of the independent effect of Lp(a) on the risk of DN

Through correlation and univariate logistic analyses, we found that among all lipid indices, Lp(a) was the most significant associated with the occurrence of DN and indicators of renal impairment. To control for confounding factors, three multivariate logistic regression models were established to analyze the independent effect of Lp(a) level on the risk of DN, in which model 1 adjusted for age and gender, model 2 adjusted for age, gender, duration of T2DM and BMI, model 3 adjusted for age, gender, duration of T2DM, BMI, SBP, DBP, HbA1c and lipid-lowering drugs usage.

**Table 3** illustrated the independent effect of Lp(a) level on the risk of DN, and its effect value is expressed as odds ratio (OR) and 95% confidence interval (CI). The magnitude of the effect value was interpreted as a relative increase in the risk of DN for each 1mg/dL increase in Lp(a) level. Model 1 showed that after adjusting for age and gender, per 1 mg/dL increase in Lp(a) level was significantly associated with a 1.128-fold increase in the risk of DN (1.128 [1.099-1.159], *P*=4.64×10^-19^). Multivariate regression analyses found that after adjusting for various confounding factors, Lp(a) level was independently positively associated with the occurrence of DN.

**Table 3:** Multivariate analysis of association between Lp(a) and DN.

Models	OR	95% CI	*P*
Model 1	1.128	1.099-1.159	**4.64×10^-19^**
Model 2	1.130	1.097-1.165	**1.95×10^-15^**
Model 3	1.115	1.079-1.152	**6.06×10^-11^**

Lp(a), Lipoprotein(a); DN, diabetic nephropathy; OR, odds ratio; CI, confidence interval; SBP, systolic blood pressure; DBP, diastolic blood pressure; BMI, body mass index; HbA1c, glycated hemoglobin. Multivariate logistic regression models were established to analyze the independent effect of Lp(a) level on the risk of DN, in which model 1 adjusted for age and gender, model 2 adjusted for age, gender, duration of T2DM and BMI, model 3 adjusted for age, gender, duration of T2DM, BMI, SBP, DBP, HbA1c and lipid-lowering drugs usage. Bold values: significant differences (P value of less than 0.05).

## 4 Discussion

The present study showed a significant association between higher Lp(a) level and increased risk of DN. One of the most important advantages of the present study is that all participants were recruited from the Department of Endocrinology, thus allowing for little heterogeneity among participants with comprehensive data available. Moreover, the sample size of this study was reasonably large, which provides a higher power to perform correlation analysis and multivariate logistic regression test.

In this study, Lp(a), ApoE, ApoB, ApoB/ApoA, TG and TC were positively correlated with DN, indicating the disturbance of lipid metabolism in DN. Among these lipid indices, there were prominent correlations between Lp(a) and indicators of renal impairment. Notably, after adjusting for various confounding factors, Lp(a) level was independently positively associated with the occurrence of DN. The mechanisms underlying the correlation between Lp(a) and renal function may be multifactorial. On one hand, the impaired renal function may cause the increased serum Lp(a) concentration (11). Patients with DN often experience a large loss of urine protein, which triggers the liver to synthesize more proteins, including lipoproteins (17). In addition to the liver, the kidney also plays a major role in fragmentation of Lp(a) (18). When kidney function is impaired, the clearance capacity of Lp(a) will decrease and serum Lp(a) level will be subsequently elevated (19). A prospective study showed that Lp(a) level decreased rapidly after renal transplantation in patients with end-stage renal disease, indicating an important metabolic role of the kidney in Lp(a) catabolism (20).

On the other hand, in line with our findings, other studies have shown Lp(a) was an independent risk factor for DN, and elevated Lp(a) level may accelerate the occurrence and progression of DN (21, 22). The involvement of Lp(a) in the development of DN may be attributed to its pro-arteriosclerotic effect. Elevated serum Lp(a) may accumulate in the glomerulus and promote arteriosclerosis in the renal arteries, as a consequence changing the glomerular filtration rate (23). In addition, the structure of Lp(a) is highly homologous to plasminogen, and Lp(a) could competently inhabit plasminogen binding to receptors, thereby inhibiting fibrinolysis, enhancing coagulation, and promoting thrombus formation (24). Besides, it has been reported that Lp(a) could increase the expression of transforming growth factor-*β*, promote fibroblast proliferation, and cause irreversible tissue fibrosis (25). Collectively, Lp(a) may serve as a target for the improvement of renal function as well as the treatment of patients with DN.

This study has several limitations. First, this study was a single-center observational study and no prospective data were available, therefore we could not conclude if the association between Lp(a) level and the occurrence of DN was causal. Second, according to the result of a meta-analysis, the association between Lp(a) and DN seems to be much stronger in the Asian population (OR: 2.29 [1.70–3.09], P < 0.001) than non-Asia population (OR: 1.24 [1.04–1.49], P = 0.02) (13).The participants in this study are all from the northwest of China, and the conclusions may not apply to other ethnic groups.

In conclusion, we show that serum Lp(a) level was significantly positively associated with the occurrence of DN, indicating that Lp(a) may have the potential as a promising target for the diagnosis and treatment of diabetic nephropathy.

## Data availability statement

All data reported in this paper will be shared by the corresponding author upon reasonable request. Requests to access these datasets should be directed to MH, mingqian_he@xjtufh.edu.cn.

## Ethics statement

The studies involving humans were approved by The Institutional Review Board at the First Affiliated Hospital of Xi’an Jiaotong University, Shaanxi, China. The studies were conducted in accordance with the local legislation and institutional requirements. The participants provided their written informed consent to participate in this study.

## Author contributions

ML: Writing – original draft. YW: Writing – original draft, Data curation. QY: Data curation, Writing – original draft. QLia: Data curation, Writing – original draft. YZ: Data curation, Writing – original draft. XW: Writing – original draft, Methodology. QLi: Methodology, Writing – original draft. WQ: Supervision, Writing – review & editing. JY: Supervision, Writing – review & editing. BS: Supervision, Writing – review & editing. MH: Supervision, Writing – review & editing.
